# Kinetic analysis and optimisation of ^18^F-rhPSMA-7.3 PET imaging of prostate cancer

**DOI:** 10.1007/s00259-021-05346-8

**Published:** 2021-04-12

**Authors:** Simona Malaspina, Vesa Oikonen, Anna Kuisma, Otto Ettala, Kalle Mattila, Peter J. Boström, Heikki Minn, Kari Kalliokoski, Ernst J. Postema, Matthew P. Miller, Mika Scheinin

**Affiliations:** 1 Turku PET Centre, University of Turku and Turku University Hospital, Turku, Finland; 2 Department of Oncology, University of Turku and Turku University Hospital, Turku, Finland; 3 Department of Urology, University of Turku and Turku University Hospital, Turku, Finland; 4 Blue Earth Diagnostics Ltd, Oxford, UK; 5 Clinical Research Services Turku – CRST Ltd, Turku, Finland

**Keywords:** ^18^F, Kinetics, PET/CT, PSMA, rhPSMA

## Abstract

**Purpose:**

This phase 1 open-label study evaluated the uptake kinetics of a novel theranostic PET radiopharmaceutical, ^18^F-rhPSMA-7.3, to optimise its use for imaging of prostate cancer.

**Methods:**

Nine men, three with high-risk localised prostate cancer, three with treatment-naïve hormone-sensitive metastatic disease and three with castration-resistant metastatic disease, underwent dynamic 45-min PET scanning of a target area immediately post-injection of 300 MBq ^18^F-rhPSMA-7.3, followed by two whole-body PET/CT scans acquired from 60 and 90 min post-injection. Volumes of interest (VoIs) corresponding to prostate cancer lesions and reference tissues were recorded. Standardised uptake values (SUV) and lesion-to-reference ratios were calculated for 3 time frames: 35–45, 60–88 and 90–118 min. Net influx rates (K_i_) were calculated using Patlak plots.

**Results:**

Altogether, 44 lesions from the target area were identified. Optimal visual lesion detection started 60 min post-injection. The ^18^F-rhPSMA-7.3 signal from prostate cancer lesions increased over time, while reference tissue signals remained stable or decreased. The mean (SD) SUV (g/mL) at the 3 time frames were 8.4 (5.6), 10.1 (7) and 10.6 (7.5), respectively, for prostate lesions, 11.2 (4.3), 13 (4.8) and 14 (5.2) for lymph node metastases, and 4.6 (2.6), 5.7 (3.1) and 6.4 (3.5) for bone metastases. The mean (SD) lesion-to-reference ratio increases from the earliest to the 2 later time frames were 40% (10) and 59% (9), respectively, for the prostate, 65% (27) and 125% (47) for metastatic lymph nodes and 25% (19) and 32% (30) for bone lesions. Patlak plots from lesion VoIs signified almost irreversible uptake kinetics. K_i_, SUV and lesion-to-reference ratio estimates showed good agreement.

**Conclusion:**

^18^F-rhPSMA-7.3 uptake in prostate cancer lesions was high. Lesion-to-background ratios increased over time, with optimal visual detection starting from 60 min post-injection. Thus, ^18^F-rhPSMA-7.3 emerges as a very promising PET radiopharmaceutical for diagnostic imaging of prostate cancer.

**Trial Registration:**

NCT03995888 (24 June 2019).

**Supplementary Information:**

The online version contains supplementary material available at 10.1007/s00259-021-05346-8.

## Introduction

^18^F-rhPSMA-7.3 is a prostate specific membrane antigen (PSMA)-targeted radiopharmaceutical in development for the imaging of patients with prostate cancer. ^18^F-labelled PSMA PET radiopharmaceuticals are increasingly used in preference to their ^68^Ga-labelled counterparts due to their favourable features, which include a longer half-life, lower positron range and larger batch production [1]. ^18^F-rhPSMA-7.3 is the lead compound of a novel series of radiohybrid PSMA (rhPSMA) radiopharmaceuticals. Further to labelling with ^18^F for imaging purposes, the agent can be labelled with radiometals for use as a therapeutic agent for patients with prostate cancer [2]. ^18^F-rhPSMA-7.3 was selected as the lead rhPSMA compound for clinical development based on preclinical assessments [3]. ^18^F-rhPSMA-7.3 is a single diastereoisomer form of ^18^F-rhPSMA-7, which has been shown to have good diagnostic efficacy in patients with primary and recurrent prostate cancer [4–6].

Prior to the start of this investigation, ^18^F-rhPSMA-7.3 was administered to six healthy volunteers to evaluate its safety, biodistribution in healthy organs and radiation dosimetry [7]. In the present phase 1 open-label study, we report the results of an evaluation of the uptake kinetics of this novel radiopharmaceutical in primary prostate tumours, local recurrence, lymph node metastases and bone metastases in patients with newly diagnosed high-risk localised prostate cancer, in treatment-naïve patients with hormone-sensitive metastatic disease and in patients with castration-resistant metastatic disease. The safety and lesion detectability results from the current study will be presented separately.

## Material and methods

### Subjects

Participants were recruited from three distinct patient populations (referred to as Cohorts A–C). All participants had histologically confirmed adenocarcinoma of the prostate without neuroendocrine differentiation or small cell features.

Cohort A comprised patients with newly diagnosed, high-risk prostate cancer as defined by the NCCN Guidelines [8] who were scheduled for radical prostatectomy. Cohort B consisted of patients who had newly diagnosed, treatment-naïve hormone-sensitive metastatic prostate cancer. Patients in Cohort C had castration-resistant metastatic prostate cancer [9], defined as a level of serum testosterone <1.7 nmol/Lplus biochemical progression (three consecutive rises in PSA 1 week apart) or radiological progression (appearance of new lesions using RECIST criteria [10]), while receiving androgen-deprivation therapy. The metastatic status in both Cohort B and C patients was defined as a quantifiable number or a high likelihood of metastases documented by standard-of-care imaging (computed tomography [CT] and/or bone scintigraphy) performed in the 12-week period preceding enrolment into this trial.

Between October 18, 2019 and March 17, 2020, 10 patients were assigned to these groups. One Cohort C patient’s PET scan was deemed not to be evaluable for the purposes of this kinetic analysis according to the predetermined study evaluability criteria as the target region with documented disease was outside of the region scanned for kinetic evaluation. The remaining nine evaluable subjects were assigned to Cohorts A–C, with three patients in each.

### Radiopharmaceutical preparation

^18^F-rhPSMA-7.3 was produced on site at Turku PET Centre using a single-use cassette-based proprietary automated synthesis platform for radiolabelling, purification and formulation (Scintomics GRP, Scintomics GmbH, Fürstenfeldbruck, Germany), and using an in-house remotely operated sterile filtration device for aseptic filling, in accordance with GMP and Turku PET Centre’s standard procedures.

### Imaging procedures

Patients were requested not to eat for at least 4 h before the administration of ^18^F-rhPSMA-7.3 but drinking of water was allowed and encouraged. Patients were encouraged to void just before ^18^F-rhPSMA-7.3 was administered and between Scans 1 and 2 (detailed below). Two i.v. cannulae were positioned, one in each arm. Diuretics were not administered for the purposes of imaging.

Scans were conducted using a GE Discovery MI PET/CT scanner (GE Healthcare, Milwaukee, WI, USA). Patients underwent a 45-min dynamic scan (Scan 1) starting at the time of ^18^F-rhPSMA-7.3 administration and two whole-body 28-min PET scans from mid-thigh to vertex (7 bed positions at 4 min/bed), starting at 60 min (Scan 2) and 90 min (Scan 3) post-injection. The first scan was performed in list mode and the target region was based on the target lesion for each patient: for Cohort A patients this was the prostate gland and for Cohort B and C patients it was the most relevant lesion identified on standard-of-care imaging. In case of multiple metastases, the target area that included the largest number of lesions was selected. Care was taken to include at least one bone lesion and one lymph node metastasis, when both present. Moreover, when possible, the area that included the thoracic or abdominal aorta was chosen.

A vacuum mattress was used to position the subjects on the PET imaging bed and to ensure the same positioning after the break between dynamic and whole-body scans. Low-dose CT scans (one of the target area and one from vertex to mid-thigh) were acquired for attenuation correction and anatomic correlation. The CT acquisition parameters were: tube potential 120 kV, tube current 10–120 mA, noise index 30, resulting in an effective dose of 1.4 mSv for the target area and 5.3 mSv for the scan from vertex to mid-thigh. The CT scan was followed by a bolus injection of ^18^F-rhPSMA-7.3.

The patients received a target radioactivity of 300 MBq (±10%) of ^18^F-rhPSMA-7.3, as informed by dosimetry data from healthy volunteers [7]. An administered activity of 300 MBq would result in a total effective dose of 4.2 mSv which was considered acceptable for the purposes of this investigation. All results were corrected for radiochemical decay from the time of administration. Scans were read according to the current interpretation guidelines for PSMA PET/CT [11] by one nuclear medicine physician (S.M) with 3 years of experience reading PSMA PET scans.

### In vitro radioactivity concentrations

Non-arterialised venous blood samples (2 mL) were collected starting after the administration of ^18^F-rhPSMA-7.3 and according to the following approximate sampling schedule: 20 s, 40 s, 60 s, 80 s, 100 s, 120 s, 140 s, 160 s, 180 s, 4 min, 5 min, 7.5 min, 10 min, 15 min, 20 min, 30 min, 40 min, 50 min, 59 min and 120 min post-injection. Blood samples were collected into heparinised tubes, mixed and chilled on ice. They were promptly divided into two aliquots, and one aliquot was centrifuged in a cooled (+4 °C) centrifuge at 2118 *g* for 5 min. Next, 700 μl of plasma was separated into another tube. Both plasma and whole blood radioactivity was measured.

### In vivo radioactivity concentrations from imaging data

Experienced PET researchers (K.K. and S.M.) manually drew volume of interest (VoI) regions on as many lesions and healthy reference tissues as were identified in the images of the target area. Blood pool VoI regions were drawn on large arteries, usually the abdominal aorta. If allowed by reference tissue size, VoIs were drawn over at least four mid-tissue PET planes with the help of the correlating anatomic CT image. Care was taken to avoid the surfaces of the reference tissues to prevent partial volume effects in the measurement of radioactivity. The positions of the VoIs were checked against all three PET scan sets and if any movement was observed, the VoI position was adjusted accordingly. Finally, the mean VoI radioactivity values of each lesion/reference tissue, each scan set and each subject were extracted and individual time–activity concentration curves (TACs) for each lesion/reference tissue were generated for each subject. The VoI radioactivity concentrations from the two static PET scans were corrected for the decay of ^18^F to the administration time, and the corrected radioactivity concentrations were added to the TACs of the dynamic scan to construct full 0–118 min regional TACs.

Regional TACs and venous plasma and blood TACs were converted from radioactivity concentration units (Bq/mL and kBq/mL, respectively) into standardised uptake value (SUV) units (g/mL) to enable direct comparisons of TACs between subjects.

SUV was calculated using Eq. 1:

1 $$ \mathrm{SUV}=\mathrm{Radioactivity}\ \mathrm{concentration}\left(\mathrm{kBq}/\mathrm{ml}\right)\times \frac{\mathrm{Weight}\ \mathrm{of}\ \mathrm{the}\ \mathrm{subject}\left(\mathrm{Kg}\right)}{\mathrm{Administered}\ \mathrm{activity}\ \left(\mathrm{MBq}\right)} $$

### Calculations based on the time–activity concentration curves

#### Plasma and blood TACs

To explore the relationship between the radioactivity concentrations in blood and plasma, the plasma-to-blood ratio as a function of time was calculated using the collected blood and plasma samples. The plasma-to-blood ratio remained constant (see the “Results” section) suggesting that ^18^F-rhPSMA-7.3 and any potential radioactive metabolites remain in blood plasma and do not enter the red blood cells. Thus, the blood TACs can be converted to plasma TACs using Eq. 2:

2 $$ \mathrm{Plasma}\ \mathrm{radioactivity}=\frac{\mathrm{Blood}\ \mathrm{radioactivity}}{1-\mathrm{Haematocrit}} $$

Haematocrit (i.e. the volume proportion of red blood cells in blood) was measured at screening, at baseline (−120 to −5 min, relative to injection), at 45 min, 3 h, 4 h and at 24 h post-injection (as part of the study safety laboratory evaluations). The average haematocrit value from the baseline, 45-min, and 3-h samples was used to convert PET image-based blood TACs to plasma TACs. Plasma-to-blood ratios averaged over time were calculated for each subject from the actual plasma and whole blood data, excluding the first 2 min, and these individual ratios were compared to the estimated plasma-to-blood ratios based on the haematocrit values.

The plasma and blood TACs were constructed as combinations of image-derived arterial TACs and manually sampled venous blood TACs to correct the expected discrepancies between the individual TACs that occur as a result of the time taken to reach transient equilibrium between arterial and venous plasma [12, 13]. Image-derived arterial blood pool TACs were utilised from the time of administration until the peak of each TAC, and manual sample venous TACs were utilised starting from their peak value. Because of the sparse sampling [14], the function in Eq. 3 was fitted to the combined plasma and blood TACs:

3 $$ f(t)=\left\{\begin{array}{c}0\\ {}\left[{A}_1\left(t-{T}_{ap}\right)-{A}_2-{A}_3\right]{e}^{-{\lambda}_1\left(t-{T}_{ap}\right)}+{A}_2{e}^{-{\lambda}_2\left(t-{T}_{ap}\right)}+{A}_3{e}^{-{\lambda}_3\left(t-{T}_{ap}\right)}\end{array}\right.{\displaystyle \begin{array}{c}, if\ t\le {T}_{ap}\\ {}, if\ t>{T}_{ap}\end{array}} $$

The fitted function parameters (coefficients A_1-3_, eigenvalues of the system λ_1-3_, and appearance time of radioactivity T_ap_) were used to construct smooth plasma and blood TACs.

#### Lesion-to-background ratios

Relative radioactivity uptake in possible cancer-associated lesions and in healthy reference tissues was analysed. For bone metastases, healthy bone marrow was used as the reference tissue. For lymph node metastases, combined initial imaging blood pool and venous blood was used as the reference tissue. For primary prostate tumours, skeletal muscle was used as the reference region. Lesion-to-reference ratios (SUV ratios) were calculated and plotted as a function of time, and ratios at the end of the dynamic scan (35–45 min) and during Scans 2 and 3 (60–88 min and 90–118 min, respectively) were calculated. During the dynamic scan, reference tissues were measured at the same time points as the lesions. In the late static scans with multiple bed positions, the measurement time points may have been somewhat different, but insignificantly so because of the relatively constant radioactivity uptake values, especially in the reference tissues.

#### Multiple-time graphical analysis

Multiple-time graphical analysis (MTGA) was performed for the target lesion and reference tissue data, using plasma TAC as an input function [15]. Compartmental models for the radiopharmaceutical and tissues of interest [16] have not been defined at this stage of development, and MTGAs (Patlak plot for irreversible and Logan plot for reversible uptake kinetics) are independent of the number of tissue compartments. We constructed Patlak and Logan plots from the data and fitted a line to the linearly increasing phase of the plot to determine the net influx rate (K_i_) in the case of a Patlak plot or the volume of distribution (V_T_) in the case of a Logan plot. Fractional uptake rates (FUR), related to the Patlak plot K_i_ [17], were calculated at different time points by dividing the VoI concentration by the area under the curve of the plasma TAC.

#### Software

The Carimas image analysis tool (version 2.10, Turku PET Centre, Turku, Finland) was used to measure radioactivity concentrations in target lesions and reference tissues at different time points post-injection. Data were processed using a spreadsheet program (Excel, Microsoft Corporation) and in-house software (tpcclib 0.7.6, Turku PET Centre, Turku, Finland).

### Statistical analysis

Descriptive data are presented as mean values and their standard deviations from the mean.

## Results

### Patients

Nine caucasian males with prostate cancer were included in this analysis. The participants had a mean age of 66 years (range 55 to 80 years) and a mean body mass index of 26.9 kg/m^2^ (range 21.9 to 30.6 kg/m^2^). PSA levels at the screening visit had ranges of 6.1–21 ng/mL for Cohort A, 3.9–35 ng/mL for Cohort B and 17–170 ng/mL for Cohort C. Testosterone levels for Cohort C patients before the PET scan were 0.05, 0.58 and 0.61 nmol/L, respectively. The mean administered activity of ^18^F-rhPSMA-7.3 was 301 MBq (range 284 to 322 MBq). The molar activity (GBq/μmol) for each patient at the time of injection is presented in Online Resource Table 1. The variation in injected mass is thought to have no significant effect on ^18^F-rhPSMA-7.3 biodistribution and tumour uptake [18].

A total of 44 lesions were identified and analysed in the selected target regions: 6 prostate/prostate bed lesions (4 primary and 2 recurrent tumours), 26 lymph node metastases and 12 bone metastases. In detail, within the target area, ^18^F-rhPSMA-7.3 identified lesions in the prostate gland in all Cohort A patients. One patient in Cohort C was found to have two sites of disease in the prostate bed. Lymph node lesions were found in two patients in Cohort B and in all three patients in Cohort C. Bone metastases were found in two patients in Cohort B and in one patient in Cohort C. No visceral metastases were identified. The great majority of lesions (36/44) were already confirmed by standard-of-care imaging. In patient B-03 and C-03, ^18^F-rhPSMA 7.3 PET/CT detected additional pelvic lymph nodes (1 and 3, respectively) that were smaller than the anatomical cut-off value used in CT (short axis of 8 mm), but that showed clearly pathological PSMA-uptake. Moreover, patient C-04, who already had confirmed nodal metastases, presented with four strong bone uptakes in the target area that were not clearly visualised by conventional imaging. Given also the already known metastatic status of the patients, these lesions were considered as metastatic. The lesions that were found in whole-body static scans outside of the target area of the dynamic scan will be reported in a separate publication.

### Blood and plasma data

Plasma and blood TACs were well fitted with the function specified in the “Material and methods” section. Blood radioactivity concentrations decreased rapidly after administration, as ^18^F-rhPSMA-7.3 was distributed in the blood pool and in the reference tissues, but radioactivity concentrations in blood remained above the average whole-body radioactivity concentration (SUV >1 g/ml). The average (SD) haematocrit during the PET investigations was 0.41 (0.04). The plasma-to-blood ratio was stable during the scans, with an average of 1.66 when calculated from samples collected starting from 2 min after radiopharmaceutical administration (Online Resource Fig. 1). Assuming that all radioactivity present in blood remains in the plasma compartment (Eq. 2), this plasma-to-blood ratio amounts to a haematocrit level of 0.40.

### Volume of interest time activity concentration curves

**Fig. 1:**
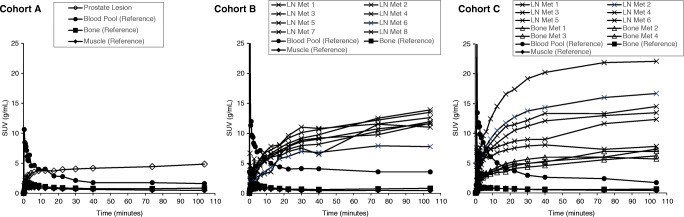
Representative SUV (g/ml) results as a function of time (min) using both the dynamic and static scan data. Left panel presents data from a patient in Cohort A, middle panel from a patient in Cohort B and right panel from a patient in Cohort C

Radioactivity concentrations, scaled for administered dose and subject weight (SUV, g/mL), as a function of time, are shown in Fig. 1 for the dynamic and static scan data combined. Target lesion uptake increased during the scanning sessions, although in less active lesions the increases were modest during the whole-body PET sessions. The mean (SD) SUVs at 35–45, 60–88 and 90–118 min were 8.4 (5.6), 10.1 (7) and 10.6 (7.5) g/ml, respectively, for prostate lesions, 11.2 (4.3), 13 (4.8) and 14 (5.2) g/ml for lymph node metastases, and 4.6 (2.6), 5.7 (3.1) and 6.4 (3.5) g/mlfor bone metastases.

The mean (SD) percentage of SUV increases from the earliest (35–45 min) to the later (60–88 and 90–118 min) scan time frames were 17% (8) and 23% (7), respectively, for the prostate, 19% (17) and 29% (20) for lymph node metastases, and 23% (12) and 40% (18) for bone lesions. Between the two later scans, the mean (SD) SUV increase in prostate, lymph node and bone lesions were 5% (3), 8% (7) and 14% (8), respectively. The absolute values of SUV for each lesion and reference tissue are shown in Online Resource Table 2.

### Lesion-to-background ratios

**Fig. 2:**
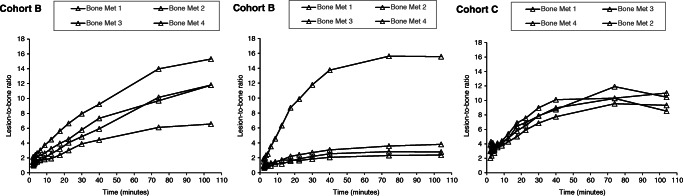
Representative lesion-to-bone-marrow ratios as a function of time. Plots displayed from patients with bone metastases (Cohort B left and middle panels, and Cohort C, right panel)

**Fig. 3:**
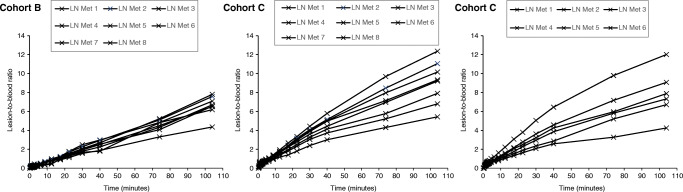
Representative lesion-to-blood (combined initial image blood pool and venous blood) ratios as a function of time. Plots displayed from patients in Cohort B (left panel) and Cohort C (middle and right panels)

**Fig. 4:**
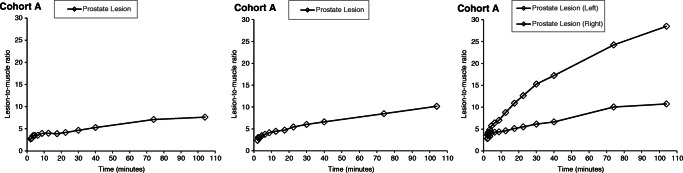
Representative lesion-to-muscle ratios as a function of time. Data shown for prostate lesions from three individual patients in Cohort A; two distinct lesions were identified in one of the patients (right panel)

The lesion-to-reference tissue ratio curves are shown in Figs. 2, 3, and 4, with healthy bone marrow, combined initial imaging blood pool and venous blood, and muscle as reference tissues, respectively. Tissue-to-blood ratios generally increased with time, suggesting a significant irreversible uptake component. The mean (SD) lesion-to-reference ratios at 35–45, 60–88 and 90–118 min were 14.5 (9.6), 20.8 (14.6) and 23.6 (16.4) for the prostate, 7 (3.5), 8.7 (4.2) and 9.1 (4.5) for lymph node metastases and 3.4 (1.4), 5.4 (2) and 7.3 (2.5) for bone lesions, respectively.

The mean (SD) lesion-to-reference ratio percentages of increases from the earliest (35–45 min) to the later (60–88 and 90–118 min) time frames were 40% (10) and 59% (9) for the prostate, 65% (27) and 125% (47) for lymph node metastases and 25% (19) and 32% (30) for bone lesions, respectively. Between the later scans, ratios increased by a mean (SD) of 14% (5), 35% (12) and 5% (10) for prostate, lymph node and bone lesions, respectively. The absolute values of ratios for each lesion/reference tissue are presented in Online Resource Table 3.

### Uptake kinetics

Tissue radioactivity concentrations and lesion-to-reference ratios increased at least until the end of the PET scanning period (118 min post-injection). The increases were not substantial after the first static scan, and optimal visual detection of primary tumours and/or metastases was achieved in the first whole-body scan, starting 60 min post-injection.

**Fig. 5:**
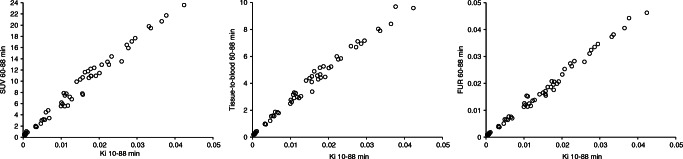
Comparison of Patlak K_i_ results with SUV, tissue-to-blood ratios and with FUR values. Left panel shows comparison with SUV. Middle plot shows comparison with tissue-to-blood ratio. Right plot shows comparison with FUR values

MTGA for irreversible uptake kinetics (Patlak plot) reached linearity about 10 min post-injection (Online Resource Fig. 2). The K_i_ values of ^18^F-rhPSMA-7.3 (calculated as the slope of the linear part of the plot) were clearly higher in suspected disease lesions than in reference tissues (Online Resource Table 4). The Patlak data suggest that ^18^F-rhPSMA-7.3 uptake kinetics in lesions and relevant reference tissues is dominated by irreversible components, but reversible components were also apparent. Since the lesion uptake kinetics of ^18^F-rhPSMA-7.3 were found to be mainly irreversible, and as all patient scans could be reliably analysed using Patlak plots, but most patient scans could not be analysed using Logan plots, the Logan plot V_T_ results are not reported here. SUVs that were calculated from the final time frame of the dynamic scan (35–45 min post-injection) and the two static scans were in good agreement with Patlak K_i_ values (Fig. 5, left panel), considering that the measured plasma input function was not used in the calculation of SUV and the usage of SUV in interindividual comparisons is based on the assumption of similar total plasma clearance. Tissue-to-blood ratios (obtained using venous blood sampling) also appeared to agree well with the Patlak K_i_ results (Fig. 5 middle panel).

FUR provides an estimate of the Patlak K_i_, using the static PET scan data and the measurement of plasma TAC from the time of ^18^F-rhPSMA-7.3 administration. FUR values also matched well with the Patlak K_i_ values (Fig. 5, right panel).

## Discussion

This phase 1 open-label study evaluated the uptake kinetics of ^18^F-rhPSMA-7.3 in nine patients with prostate cancer in order to optimise its use for PET/CT imaging.

Following administration, ^18^F-rhPSMA-7.3 uptake in the reference tissues was of a similar level to that noted in healthy volunteers [7]. We observed high uptake in prostate cancer lesions within the first hour and both the tissue radioactivity concentration and lesion-to-reference ratios increased at least until the end of the scanning sessions (118 min post-injection). This might suggest that later time points could provide more optimal imaging, as has been previously demonstrated for other PSMA-tracers [19–22]. However, according to the results of our study, the increases were not substantial after the first whole-body scan (Scan 2). Therefore, considering logistical reasons for diagnostic use in clinical practice, the optimal time for ^18^F-rhPSMA-7.3 PET for the assessment of both primary prostate tumours and metastatic lesions is between 60 and 90 min post-injection and the optimal timing to commence scanning is 60 min post-injection. In contrast with the studies mentioned above with other tracers, we have not evaluated later time points (> 2 h post-injection) in the present study. This might be a limitation, considering that this information could be useful in a radiometabolic treatment setting. However, this study was designed to investigate the uptake kinetics of rhPSMA 7.3 specifically to optimise its diagnostic use.

Radioactivity concentrations in the blood decreased rapidly after ^18^F-rhPSMA-7.3 administration as ^18^F-rhPSMA-7.3 was distributed in the blood pool and in the reference tissues. Our data show that the plasma-to-blood ratio remained constant over the scanning period and at a level implying that ^18^F-rhPSMA-7.3 remains in blood plasma and does not enter the red blood cells during the relevant time period from injection. As demonstrated by our previous studies with ^18^F-FDG [23, 24], combining venous sampling and image-based arterial/venous estimates as input functions for the TACs is a useful technique in kinetic studies. Here, combining venous blood sampling data with initial arterial data from a blood pool in the PET images improved the otherwise underestimated initial phase of the plasma TACs. Generally, if a blood pool of high enough quality can be identified from PET images, then venous sampling can be replaced with a blood pool-derived estimation from the PET image, thus removing the need for blood sampling for radioactivity analyses.

The general increase in tissue-to-blood ratios with time suggests a significant irreversible uptake component. The MTGA data further confirm this; the Patlak plots suggest that ^18^F-rhPSMA-7.3 uptake kinetics in lesions and relevant reference tissues is dominated by irreversible components, but some reversible components were also apparent. This is as expected for a radiopharmaceutical that can bind reversibly to its specific target molecule residing on cell membranes but can also be internalised into cells when bound to its receptor, and possibly slowly recirculated back to the cell surface. Downward curvature of the Patlak plot leads to K_i_ estimates that are dependent on the scan duration and on the selection of the time range used for the slope estimation from the plot. If the uptake kinetics were fully irreversible during the PET scan, the Patlak slope would be time-independent after an initial equilibration period; this would be a considerable advantage over SUV and ratio methods which can only provide time-dependent measures. The curvature of the plots prevents a bias-free, scan duration-independent assessment of the slope (net influx rate K_i_ in the case of Patlak plots). Thus, a dynamic PET protocol with blood sampling may not offer such quantitative advantages over the simpler SUV and ratio methods that would support the use of a longer and more laborious imaging protocol.

Since Patlak K_i_ estimates are dependent on the selected line fit time range, and FUR estimates on the PET time frame used for the calculations, the results are here reported from different time frames. The decreases over time suggest that extending the PET scan duration beyond 90 min may not improve the detectability of lesions, possibly even the opposite, if Patlak or FUR analyses are employed.

Taken together, the different analyses presented here provided concordant results. The findings indicate that neither dynamic PET scans nor blood sampling should be required in clinical applications of ^18^F-rhPSMA-7.3 imaging of patients with prostate cancer.

The present study is not without limitations. Although not uncommon for studies of this nature, the small number of study participants limits the conclusions that can be drawn from these data. Nevertheless, the present results have already been used to determine the optimal time window for PET imaging following administration of ^18^F-rhPSMA-7.3 in the ongoing pivotal phase 3 trials of ^18^F-rhPSMA-7.3 in newly diagnosed patients [LIGHTHOUSE; NCT04186819] and in patients with biochemical recurrence of prostate cancer [SPOTLIGHT; NCT04186845], and the results of these trials are eagerly awaited.

## Conclusions

^18^F-rhPSMA-7.3 uptake kinetics in prostate cancer lesions and relevant reference tissues are dominated by irreversible components. Uptake in prostate cancer lesions and lesion-to-background ratios increased over time, with the optimal timing for ^18^F-rhPSMA-7.3 PET imaging for the assessment of both primary prostate tumours and metastatic lesions appearing to be between 60 and 90 min post-injection. The optimal timing to commence scanning is therefore 60 min post-injection. In the clinical setting, simplified measures of ^18^F-rhPSMA-7.3 uptake are sufficient for optimal detection of prostate cancer lesions, removing the need for dynamic PET scans and/or blood sampling. Thus, ^18^F-rhPSMA-7.3 emerges as a very promising radiopharmaceutical for diagnostic imaging of prostate cancer, with possible further applications in theranostics.

## Supplementary Information

ESM 1 (PPTX 53 kb)

ESM 2 (PPTX 66 kb)

ESM 3 (DOCX 24 kb)

ESM 4 (DOCX 31 kb)

ESM 5 (DOCX 32 kb)

ESM 6 (DOCX 31 kb)

## Data Availability

Anonymised data are available from the Corresponding author on reasonable request.
