# Supplementary material for: Kinetic analysis and optimisation of 18F-rhPSMA-7.3 PET imaging of prostate cancer
Source: Eur J Nucl Med Mol Imaging. 2021 Apr 12;48(11):3723–31. doi: 10.1007/s00259-021-05346-8 (PMC8440272; doi:10.1007/s00259-021-05346-8)
Supplement: Supplementary file 1 — (PPTX 53 kb) [file 259_2021_5346_MOESM1_ESM.pptx]

## Slide 1
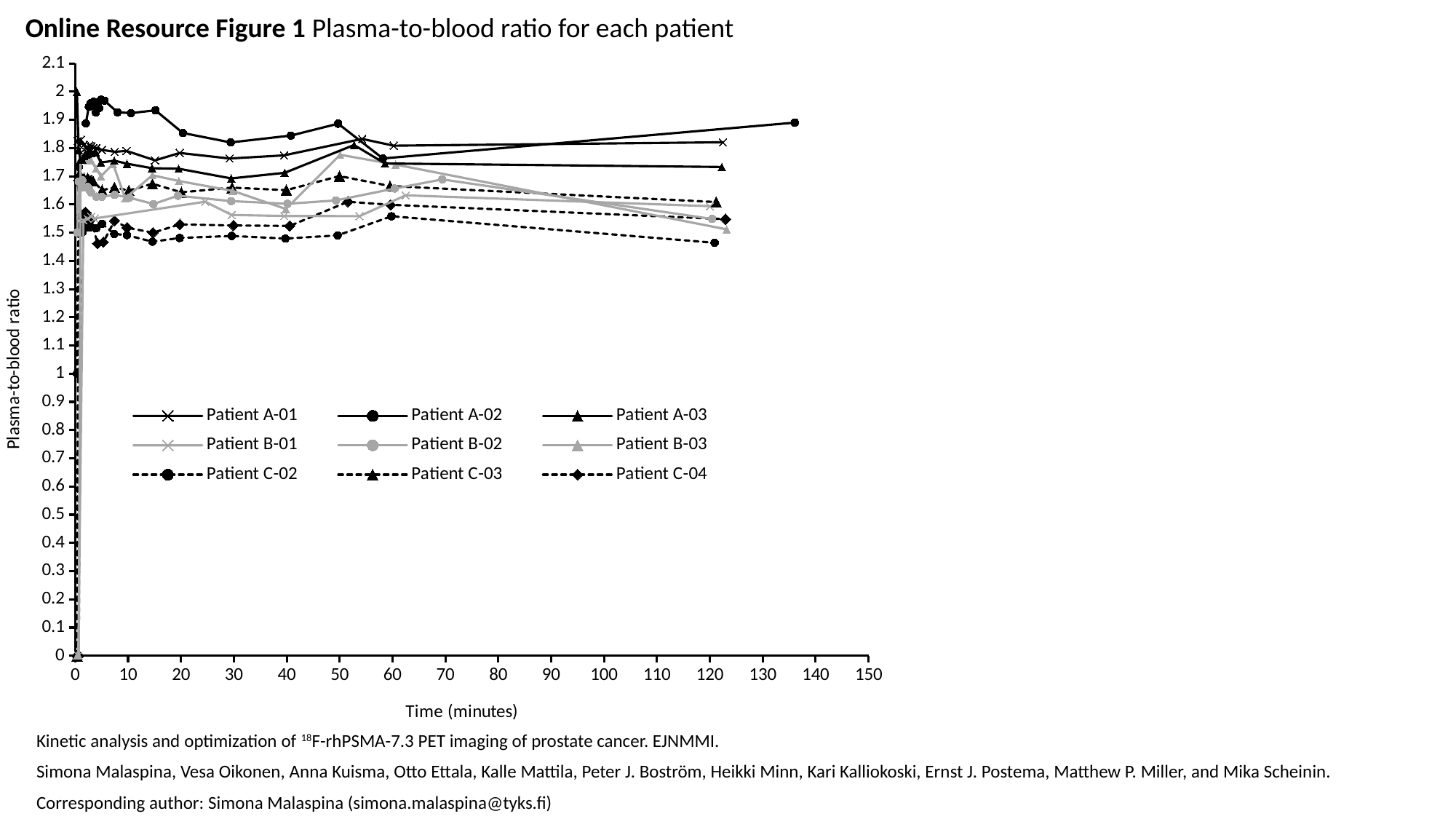

Online Resource Figure 1 Plasma-to-blood ratio for each patient
### Chart
| Category | Patient A-01 | Patient A-02 | Patient A-03 | Patient B-01 | Patient B-02 | Patient B-03 | Patient C-02 | Patient C-03 | Patient C-04 |
|---|---|---|---|---|---|---|---|---|---|Kinetic analysis and optimization of 18F-rhPSMA-7.3 PET imaging of prostate cancer. EJNMMI.
Simona Malaspina, Vesa Oikonen, Anna Kuisma, Otto Ettala, Kalle Mattila, Peter J. Boström, Heikki Minn, Kari Kalliokoski, Ernst J. Postema, Matthew P. Miller, and Mika Scheinin.
Corresponding author: Simona Malaspina (simona.malaspina@tyks.fi)
