# Supplementary material for: Kinetic analysis and optimisation of 18F-rhPSMA-7.3 PET imaging of prostate cancer
Source: Eur J Nucl Med Mol Imaging. 2021 Apr 12;48(11):3723–31. doi: 10.1007/s00259-021-05346-8 (PMC8440272; doi:10.1007/s00259-021-05346-8)
Supplement: Supplementary file 2 — (PPTX 66 kb) [file 259_2021_5346_MOESM2_ESM.pptx]

## Slide 1
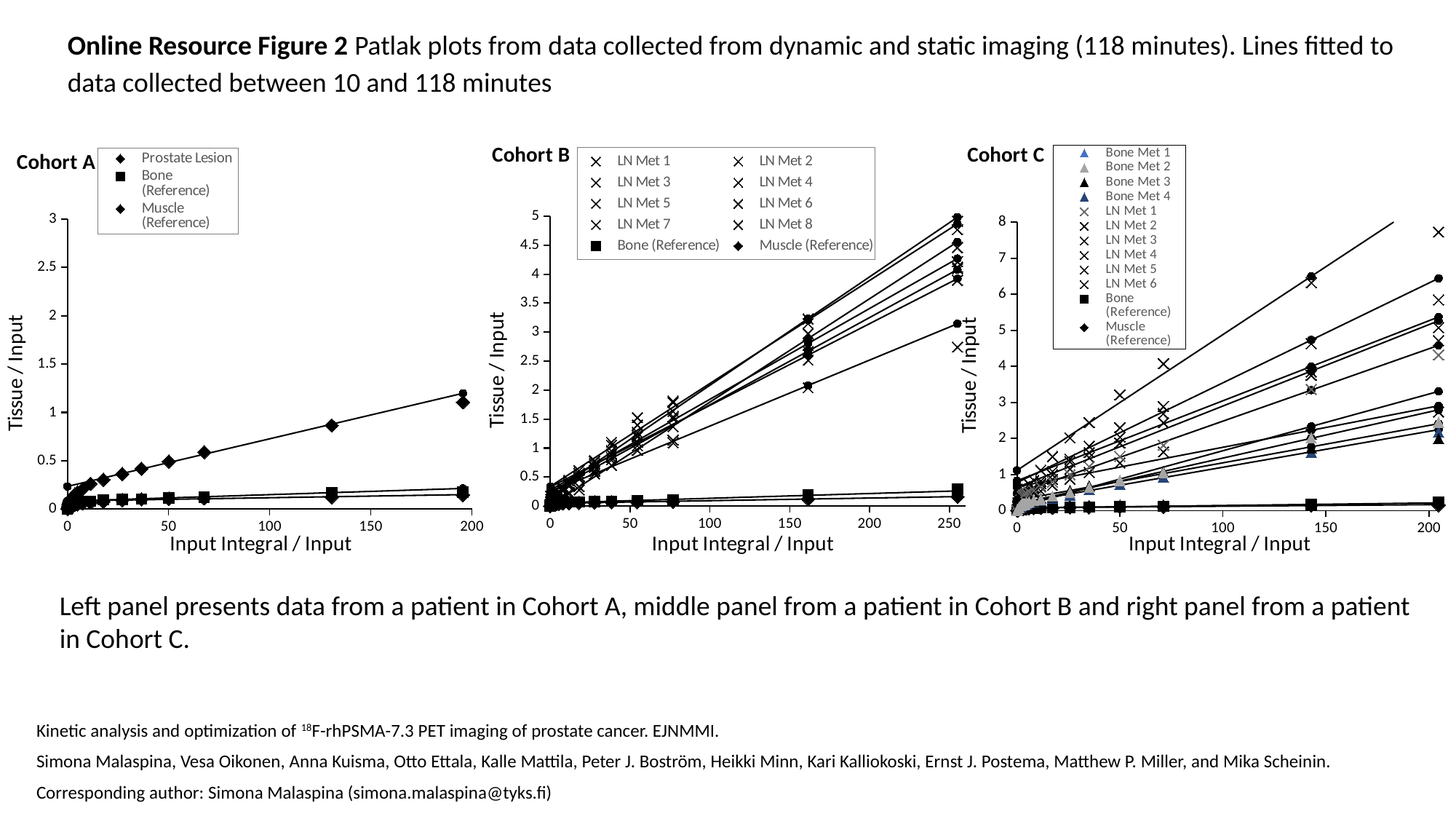

Online Resource Figure 2 Patlak plots from data collected from dynamic and static imaging (118 minutes). Lines fitted to data collected between 10 and 118 minutes
### Chart: Cohort A
| Category | Prostate Lesion | Bone (Reference) | Muscle (Reference) | | | |
|---|---|---|---|---|---|---|
### Chart: Cohort B
| Category | | LN Met 1 | LN Met 2 | | LN Met 3 | | LN Met 4 | | LN Met 5 | | LN Met 6 | | LN Met 7 | | LN Met 8 | | Bone (Reference) | | Muscle (Reference) | |
|---|---|---|---|---|---|---|---|---|---|---|---|---|---|---|---|---|---|---|---|---|
### Chart: Cohort C
| Category | Bone Met 1 | | Bone Met 2 | | Bone Met 3 | | Bone Met 4 | | LN Met 1 | | LN Met 2 | | LN Met 3 | | LN Met 4 | | LN Met 5 | | LN Met 6 | | Bone (Reference) | | Liver (Reference) | | Muscle (Reference) | |
|---|---|---|---|---|---|---|---|---|---|---|---|---|---|---|---|---|---|---|---|---|---|---|---|---|---|---|Left panel presents data from a patient in Cohort A, middle panel from a patient in Cohort B and right panel from a patient in Cohort C.
Kinetic analysis and optimization of 18F-rhPSMA-7.3 PET imaging of prostate cancer. EJNMMI.
Simona Malaspina, Vesa Oikonen, Anna Kuisma, Otto Ettala, Kalle Mattila, Peter J. Boström, Heikki Minn, Kari Kalliokoski, Ernst J. Postema, Matthew P. Miller, and Mika Scheinin.
Corresponding author: Simona Malaspina (simona.malaspina@tyks.fi)
