# Supplementary material for: Kinetic analysis and optimisation of 18F-rhPSMA-7.3 PET imaging of prostate cancer
Source: Eur J Nucl Med Mol Imaging. 2021 Apr 12;48(11):3723–31. doi: 10.1007/s00259-021-05346-8 (PMC8440272; doi:10.1007/s00259-021-05346-8)
Supplement: Supplementary file 3 — (DOCX 24 kb) [file 259_2021_5346_MOESM3_ESM.docx]

**Online Resource Table 1**

| Molar activity at the time of injection | |
| --- | --- |
| Patient | Molar activity (GBq/µmol) |
| A-01 | 45.5466 |
| A-02 | 39.0245 |
| A-03 | 44.9167 |
| B-01 | 64.5382 |
| B-02 | 56.0477 |
| B-03 | 43.8495 |
| C-02 | 42.3231 |
| C-03 | 38.4733 |
| C-04 | 59.2139 |
