# Supplementary material for: Kinetic analysis and optimisation of 18F-rhPSMA-7.3 PET imaging of prostate cancer
Source: Eur J Nucl Med Mol Imaging. 2021 Apr 12;48(11):3723–31. doi: 10.1007/s00259-021-05346-8 (PMC8440272; doi:10.1007/s00259-021-05346-8)
Supplement: Supplementary file 4 — (DOCX 31 kb) [file 259_2021_5346_MOESM4_ESM.docx]

**Online Resource Table 2**

| **SUV (g/ml) of lesions and reference tissues at the three study timepoints** | | | | |
| --- | --- | --- | --- | --- |
| Patient | Lesion or reference tissue | 35–45 min | 60–88 min | 90–118 min |
| A-01 | Reference tissue: muscle | 0.521 | 0.4341 | 0.406 |
| A-01 | Tumour: prostate | 2.7556 | 3.0089 | 3.0976 |
| A-02 | Reference tissue: muscle | 0.6337 | 0.5299 | 0.478 |
| A-02 | Tumour: prostate | 4.1856 | 4.468 | 4.8611 |
| A-03 | Reference tissue: muscle | 0.5767 | 0.49423 | 0.44411 |
| A-03 | Tumour: prostate (L) | 3.82 | 4.7922 | 4.7685 |
| A-03 | Tumour: prostate (R) | 9.9315 | 11.787 | 12.659 |
| B-01 | Metastasis: bone (1) | 5.839 | 7.7846 | 8.8348 |
| B-01 | Metastasis: bone (2) | 4.6406 | 5.536 | 6.7978 |
| B-01 | Metastasis: bone (3) | 3.7448 | 5.6444 | 6.8078 |
| B-01 | Metastasis: bone (4) | 2.801 | 3.4187 | 3.7876 |
| B-01 | Reference tissue: bone | 0.6328 | 0.5726 | 0.577 |
| B-02 | Metastasis: lymph node (1) | 10.807 | 11.597 | 12.621 |
| B-02 | Metastasis: lymph node (2) | 10.64 | 12.195 | 13.498 |
| B-02 | Metastasis: lymph node (3) | 9.0271 | 10.673 | 11.598 |
| B-02 | Metastasis: lymph node (4) | 9.7569 | 12.39 | 13.907 |
| B-02 | Metastasis: lymph node (5) | 6.5162 | 10.944 | 11.022 |
| B-02 | Metastasis: lymph node (6) | 6.8035 | 7.7764 | 7.7644 |
| B-02 | Metastasis: lymph node (7) | 8.1828 | 10.308 | 11.931 |
| B-02 | Metastasis: lymph node (8) | 9.2053 | 9.9201 | 11.664 |
| B-02 | Reference: blood | 3.6287 | 2.3921 | 1.782 |
| B-03 | Metastasis: bone (1) | 11.235 | 13.534 | 15.452 |
| B-03 | Metastasis: bone (2) | 2.5069 | 3.1267 | 3.7788 |
| B-03 | Metastasis: bone (3) | 1.6881 | 2.0084 | 2.354 |
| B-03 | Metastasis: bone (4) | 2.0795 | 2.4496 | 2.7676 |
| B-03 | Reference tissue: bone | 0.81742 | 0.87761 | 0.99358 |
| B-03 | Metastasis: lymph node | 2.4364 | 3.1759 | 4.0608 |
| B-03 | Reference: blood | 2.7979 | 2.0302 | 1.5792 |
| C-02 | Metastasis: lymph node (1) | 18.795 | 23.581 | 25.65 |
| C-02 | Metastasis: lymph node (2) | 16.701 | 20.688 | 22.938 |
| C-02 | Metastasis: lymph node (3) | 14.408 | 17.095 | 19.107 |
| C-02 | Metastasis: lymph node (4) | 16.277 | 19.46 | 21.101 |
| C-02 | Metastasis: lymph node (5) | 16.16 | 17.67 | 19.385 |
| C-02 | Metastasis: lymph node (6) | 9.7627 | 10.605 | 11.266 |
| C-02 | Metastasis: lymph node (7) | 13.423 | 14.416 | 16.417 |
| C-02 | Metastasis: lymph node (8) | 12.103 | 12.93 | 14.112 |
| C-02 | Reference: blood | 3.6287 | 2.3921 | 1.782 |
| C-03 | Metastasis: lymph node (1) | 6.5094 | 10.958 | 11.831 |
| C-03 | Metastasis: lymph node (2) | 9.6139 | 10.916 | 11.51 |
| C-03 | Metastasis: lymph node (3) | 5.9359 | 7.1893 | 6.7024 |
| C-03 | Reference: blood | 3.1266 | 2.4765 | 2.0644 |
| C-03 | Recurrent tumour: prostate | 15.43 | 19.782 | 20.656 |
| C-03 | Reference tissue: muscle | 0.5812 | 0.48471 | 0.4545 |
| C-03 | Recurrent tumour: prostate | 14.211 | 16.47 | 17.756 |
| C-04 | Metastasis: lymph node (1) | 9.0068 | 11.447 | 12.345 |
| C-04 | Metastasis: lymph node (2) | 14.321 | 15.921 | 16.694 |
| C-04 | Metastasis: lymph node (3) | 8.0753 | 7.4585 | 7.8215 |
| C-04 | Metastasis: lymph node (4) | 12.098 | 12.957 | 13.47 |
| C-04 | Metastasis: lymph node (5) | 13.355 | 13.455 | 14.505 |
| C-04 | Metastasis: lymph node (6) | 20.229 | 21.731 | 22.091 |
| C-04 | Reference: blood | 3.1361 | 2.2336 | 1.8397 |
| C-04 | Metastasis: bone (1) | 5.9984 | 6.1831 | 7.3665 |
| C-04 | Metastasis: bone (2) | 5.1922 | 6.7868 | 6.9901 |
| C-04 | Metastasis: bone (3) | 5.3246 | 5.903 | 5.698 |
| C-04 | Metastasis: bone (4) | 4.6023 | 5.5576 | 6.2307 |
| C-04 | Reference tissue: bone | 0.59379 | 0.59549 | 0.66821 |
