# Supplementary material for: Kinetic analysis and optimisation of 18F-rhPSMA-7.3 PET imaging of prostate cancer
Source: Eur J Nucl Med Mol Imaging. 2021 Apr 12;48(11):3723–31. doi: 10.1007/s00259-021-05346-8 (PMC8440272; doi:10.1007/s00259-021-05346-8)
Supplement: Supplementary file 5 — (DOCX 32 kb) [file 259_2021_5346_MOESM5_ESM.docx]

**Online Resource Table 3**

| **SUV lesion-to-background ratios stratified by study timepoints** | | | | |
| --- | --- | --- | --- | --- |
| Patient | Lesion | 35–45 min | 60–88 min | 90–118 min |
| Lesion-to-blood ratio | | | | |
| B-02 | Metastasis: lymph node (1) | 2.9782 | 4.8480 | 7.0825 |
| B-02 | Metastasis: lymph node (2) | 2.9322 | 5.0980 | 7.5746 |
| B-02 | Metastasis: lymph node (3) | 2.4877 | 4.4618 | 6.5084 |
| B-02 | Metastasis: lymph node (4) | 2.6888 | 5.1795 | 7.8042 |
| B-02 | Metastasis: lymph node (5) | 1.7957 | 4.5751 | 6.1852 |
| B-02 | Metastasis: lymph node (6) | 1.8749 | 3.2509 | 4.3571 |
| B-02 | Metastasis: lymph node (7) | 2.2550 | 4.3092 | 6.6953 |
| B-02 | Metastasis: lymph node (8) | 2.5368 | 4.1470 | 6.5455 |
| B-03 | Metastasis: lymph node | 0.8708 | 1.5643 | 2.5714 |
| C-02 | Metastasis: lymph node (1) | 5.7913 | 9.5601 | 12.3585 |
| C-02 | Metastasis: lymph node (2) | 5.1461 | 8.3873 | 11.0518 |
| C-02 | Metastasis: lymph node (3) | 4.4395 | 6.9306 | 9.2060 |
| C-02 | Metastasis: lymph node (4) | 5.0154 | 7.8894 | 10.1667 |
| C-02 | Metastasis: lymph node (5) | 4.9794 | 7.1637 | 9.3399 |
| C-02 | Metastasis: lymph node (6) | 3.0082 | 4.2994 | 5.4281 |
| C-02 | Metastasis: lymph node (7) | 4.1360 | 5.8445 | 7.9099 |
| C-02 | Metastasis: lymph node (8) | 3.7293 | 5.2420 | 6.7993 |
| C-03 | Metastasis: lymph node (1) | 2.0819 | 4.4248 | 5.7310 |
| C-03 | Metastasis: lymph node (2) | 3.0749 | 4.4078 | 5.5755 |
| C-03 | Metastasis: lymph node (3) | 1.8985 | 2.9030 | 3.2467 |
| C-04 | Metastasis: lymph node (1) | 2.8720 | 5.1249 | 6.7103 |
| C-04 | Metastasis: lymph node (2) | 4.5665 | 7.1280 | 9.0743 |
| C-04 | Metastasis: lymph node (3) | 2.5749 | 3.3392 | 4.2515 |
| C-04 | Metastasis: lymph node (4) | 3.8577 | 5.8009 | 7.3218 |
| C-04 | Metastasis: lymph node (5) | 4.2585 | 6.0239 | 7.8844 |
| C-04 | Metastasis: lymph node (6) | 6.4504 | 9.7291 | 12.0079 |
| Lesion-to-bone ratio | | | | |
| B-01 | Metastasis: bone (1) | 9.2267 | 13.646 | 15.311 |
| B-01 | Metastasis: bone (2) | 7.3331 | 9.6923 | 11.781 |
| B-01 | Metastasis: bone (3) | 5.9175 | 9.9023 | 11.798 |
| B-01 | Metastasis: bone (4) | 4.4261 | 5.9885 | 6.5642 |
| B-03 | Metastasis: bone (1) | 13.744 | 15.41 | 15.552 |
| B-03 | Metastasis: bone (2) | 3.0669 | 3.5557 | 3.8032 |
| B-03 | Metastasis: bone (3) | 2.0652 | 2.2858 | 2.3692 |
| B-03 | Metastasis: bone (4) | 2.544 | 2.7899 | 2.7854 |
| C-04 | Metastasis: bone (1) | 10.102 | 10.373 | 11.024 |
| C-04 | Metastasis: bone (2) | 8.7441 | 11.417 | 10.461 |
| C-04 | Metastasis: bone (3) | 8.9671 | 9.9375 | 8.5272 |
| C-04 | Metastasis: bone (4) | 7.7507 | 9.3355 | 9.3245 |
| Lesion-to-muscle ratio | | | | |
| A-01 | Tumour: prostate | 5.2888 | 6.9722 | 7.6292 |
| A-02 | Tumour: prostate | 6.6051 | 8.4889 | 10.169 |
| A-03 | Tumour: prostate (L) | 6.6243 | 9.7630 | 10.737 |
| A-03 | Tumour: prostate (R) | 17.222 | 24.020 | 28.504 |
| C-03 | Recurrent tumour: prostate | 26.548 | 41.16 | 45.448 |
| C-03 | Recurrent tumour: prostate | 24.451 | 34.221 | 39.068 |
