# Supplementary material for: Kinetic analysis and optimisation of 18F-rhPSMA-7.3 PET imaging of prostate cancer
Source: Eur J Nucl Med Mol Imaging. 2021 Apr 12;48(11):3723–31. doi: 10.1007/s00259-021-05346-8 (PMC8440272; doi:10.1007/s00259-021-05346-8)
Supplement: Supplementary file 6 — (DOCX 31 kb) [file 259_2021_5346_MOESM6_ESM.docx]

**Online Resource Table 4**

| **Online Resource Table 3: Patlak plot K_i_ (1/min) stratified by study timepoints** | | | | |
| --- | --- | --- | --- | --- |
| Patient | Lesion or reference tissue | 10–45 min | 10–88 min | 10–118 min |
| A-01 | Reference tissue: bone | 0.000585 | 0.000672 | 0.000485 |
| A-01 | Reference tissue: muscle | 0.000641 | 0.000339 | 0.000319 |
| A-01 | Tumour: prostate | 0.005676 | 0.004929 | 0.004488 |
| A-02 | Reference tissue: bone | 0.000814 | 0.00073 | 0.000791 |
| A-02 | Reference tissue: muscle | 0.000698 | 0.00041 | 0.000311 |
| A-02 | Tumour: prostate | 0.007508 | 0.005997 | 0.005654 |
| A-03 | Reference tissue: bone | 0.001293 | 0.000867 | 0.000916 |
| A-03 | Reference tissue: muscle | 0.000599 | 0.000367 | 0.000303 |
| A-03 | Tumour: prostate (L) | 0.006336 | 0.006596 | 0.005658 |
| A-03 | Tumour: prostate (R) | 0.020197 | 0.017098 | 0.01557 |
| B-01 | Reference tissue: bone | 0.000957 | 0.000697 | 0.000663 |
| B-01 | Reference tissue: muscle | 0.000856 | 0.000587 | 0.000516 |
| B-01 | Metastasis: bone (1) | 0.014964 | 0.015602 | 0.015274 |
| B-01 | Metastasis: bone (2) | 0.012318 | 0.010959 | 0.011557 |
| B-01 | Metastasis: bone (3) | 0.010012 | 0.011792 | 0.012173 |
| B-01 | Metastasis: bone (4) | 0.007652 | 0.006881 | 0.00651 |
| B-02 | Reference tissue: bone | 0.000555 | 0.00082 | 0.000936 |
| B-02 | Reference tissue: muscle | 0.000305 | 0.000455 | 0.000443 |
| B-02 | Metastasis: lymph node (1) | 0.021297 | 0.016257 | 0.015774 |
| B-02 | Metastasis: lymph node (2) | 0.020895 | 0.017767 | 0.017408 |
| B-02 | Metastasis: lymph node (3) | 0.017295 | 0.015659 | 0.015039 |
| B-02 | Metastasis: lymph node (4) | 0.018986 | 0.018699 | 0.01845 |
| B-02 | Metastasis: lymph node (5) | 0.013326 | 0.017834 | 0.015433 |
| B-02 | Metastasis: lymph node (6) | 0.01352 | 0.011379 | 0.00993 |
| B-02 | Metastasis: lymph node (7) | 0.015158 | 0.015041 | 0.015537 |
| B-02 | Metastasis: lymph node (8) | 0.018807 | 0.014098 | 0.014821 |
| B-03 | Reference tissue: bone | 0.001069 | 0.001147 | 0.00133 |
| B-03 | Reference tissue: muscle | 0.000752 | 0.000551 | 0.00062 |
| B-03 | Metastasis: bone (1) | 0.030011 | 0.02593 | 0.02559 |
| B-03 | Metastasis: bone (2) | 0.006022 | 0.005717 | 0.006146 |
| B-03 | Metastasis: bone (3) | 0.003048 | 0.003298 | 0.003611 |
| B-03 | Metastasis: bone (4) | 0.005572 | 0.004661 | 0.004552 |
| B-03 | Metastasis: lymph node | 0.005113 | 0.005372 | 0.006387 |
| C-02 | Reference tissue: bone | 0.001180 | 0.000732 | 0.000751 |
| C-02 | Reference tissue: muscle | 0.000906 | 0.000266 | 0.000225 |
| C-02 | Metastasis: lymph node (1) | 0.044452 | 0.042368 | 0.039124 |
| C-02 | Metastasis: lymph node (2) | 0.039658 | 0.036472 | 0.034451 |
| C-02 | Metastasis: lymph node (3) | 0.032311 | 0.028609 | 0.027615 |
| C-02 | Metastasis: lymph node (4) | 0.037759 | 0.033589 | 0.031077 |
| C-02 | Metastasis: lymph node (5) | 0.037823 | 0.029486 | 0.027655 |
| C-02 | Metastasis: lymph node (6) | 0.021764 | 0.017303 | 0.01576 |
| C-02 | Metastasis: lymph node (7) | 0.030825 | 0.023288 | 0.02298 |
| C-02 | Metastasis: lymph node (8) | 0.027461 | 0.02069 | 0.019455 |
| C-02 | Reference tissue: bone | 0.00118 | 0.000732 | 0.000751 |
| C-03 | Reference tissue: bone | 0.000774 | 0.000626 | 0.000694 |
| C-03 | Reference tissue: muscle | 0.000554 | 0.000278 | 0.000262 |
| C-03 | Metastasis: lymph node (1) | 0.01296 | 0.019159 | 0.017625 |
| C-03 | Metastasis: lymph node (2) | 0.022614 | 0.017951 | 0.01583 |
| C-03 | Metastasis: lymph node (3) | 0.013547 | 0.012278 | 0.009402 |
| C-03 | Recurrent tumour: prostate | 0.035164 | 0.033113 | 0.029024 |
| C-03 | Recurrent tumour: prostate | 0.033723 | 0.027277 | 0.024671 |
| C-04 | Reference tissue: bone | 0.000701 | 0.000688 | 0.000783 |
| C-04 | Reference tissue: muscle | 0.000485 | 0.000427 | 0.000372 |
| C-04 | Metastasis: lymph node (1) | 0.018853 | 0.020092 | 0.018955 |
| C-04 | Metastasis: lymph node (2) | 0.033453 | 0.027693 | 0.0251 |
| C-04 | Metastasis: lymph node (3) | 0.01723 | 0.010989 | 0.01026 |
| C-04 | Metastasis: lymph node (4) | 0.028969 | 0.02259 | 0.020214 |
| C-04 | Metastasis: lymph node (5) | 0.030751 | 0.022207 | 0.020935 |
| C-04 | Metastasis: lymph node (6) | 0.047611 | 0.037682 | 0.032951 |
| C-04 | Metastasis: bone (1) | 0.014093 | 0.010093 | 0.010698 |
| C-04 | Metastasis: bone (2) | 0.012218 | 0.01279 | 0.011288 |
| C-04 | Metastasis: bone (3) | 0.012156 | 0.010367 | 0.008574 |
| C-04 | Metastasis: bone (4) | 0.010791 | 0.00997 | 0.009693 |
